# Adaptation of Essential Care for Every Baby educational program to improve infant outcomes in the context of Zika

**DOI:** 10.1186/s12887-022-03710-7

**Published:** 2022-11-21

**Authors:** Kera McNelis, Nina Prasanphanich, Susanne P. Martin-Herz, Terrell Carter, Hannah Foehringer Merchant, Janna Patterson, Salwan Hager, Tamar Chitashvili, Shivon Belle Jarvis, Beena D. Kamath-Rayne

**Affiliations:** 1 Department of Pediatrics, Cincinnati Children’s Hospital Medical Center, 3333 Burnet Avenue, MLC 7009, Cincinnati, OH 45229-3026 USA; 2 University of Cincinnati College of Medicine, Cincinnati, OH USA; 3 Department of Pediatrics, University of California, San Francisco, CA USA; 4 American Academy of Pediatrics, Itasca, IL USA; 5 University Research Co., LLC, Chevy Chase, MD USA; 6 Paediatric Department, Mount St. John’s Medical Centre, St. John’s, Antigua and Barbuda

**Keywords:** Congenital syndrome associated with Zika, Essential Care for Every Baby, Essential newborn care, Newborn, Zika

## Abstract

**Background:**

The outbreak and ongoing transmission of Zika virus provided an opportunity to strengthen essential newborn care and early childhood development systems through collaboration with the US Agency for International Development Applying Science to Strengthen and Improve Systems (USAID ASSIST). The objective was to create a system of sustainable training dissemination which improves newborn care-related quality indicators in the context of Zika.

**Methods:**

From 2018–19, USAID ASSIST supported a series of technical assistance visits by the American Academy of Pediatrics (AAP) in four Caribbean countries to strengthen the clinical capacity in care of children potentially affected by Zika through dissemination of Essential Care for Every Baby (ECEB), teaching QI methodology, coaching visits, and development of clinical care guidelines. ECEB was adapted to emphasize physical exam findings related to Zika. The first series of workshops were facilitated by AAP technical advisors and the second series were facilitated by the newly trained local champions. Quality of care was monitored with performance indicators at 134 health facilities.

**Results:**

A repeated measures (pre-post) ANOVA was conducted, revealing significant pre-post knowledge gains [*F*(1) = 197.9, *p* < 0.001] on knowledge check scores. Certain performance indicators related to ECEB practices demonstrated significant changes and midline shift on the run chart in four countries.

**Conclusion:**

ECEB can be adapted to incorporate important local practices, causes of neonatal morbidity and mortality, and differing healthcare system structures, which, as one part of a larger technical assistance package, leads to improved performance of health systems.

**Supplementary Information:**

The online version contains supplementary material available at 10.1186/s12887-022-03710-7.

## Background

The UN Sustainable Development Goals (SDG) target reductions in neonatal mortality rate and include ending preventable deaths of newborns and children under 5 years of age [1]. A collaborative toolkit has been developed to support the implementation of the *Global Strategy for Women’s, Children’s and Adolescent’s Health* and accelerate the health-related Development Goals. However, there is currently a lack of evidence on successful implementation strategies to improve newborn care across systems of practice [2, 3]. The outbreak and ongoing transmission of Zika virus in Latin America and the Caribbean since 2016 provided an opportunity to strengthen essential newborn care and early childhood development systems in Zika-affected countries through donor assistance. Beginning in July 2018, the US Agency for International Development Applying Science to Strengthen and Improve Systems (USAID ASSIST) Project began working with Ministries of Health (MOH) to provide short-term technical assistance in four Eastern and Southern Caribbean countries (Antigua and Barbuda, St. Vincent and the Grenadines, St. Kitts and Nevis, and Dominica) to improve early detection, care and support of children and families potentially affected by Zika [4]. Full description of this technical assistance program is described elsewhere [4].

Data obtained prior to the onset of the project indicated that the total number of suspected cases of Zika in these countries ranged from 508 to 1154 from 2015 to January 2018, and 5–17 confirmed cases in pregnant women per country, indicating at least 1% of pregnancies were affected by Zika infection. A case of Zika was considered confirmed if the patient tested positive by IgM or PCR. As uniform screening of pregnant mothers did not exist during the period of active transmission, the possibility of underdiagnoses of Zika infection was significant [5]. The median neonatal mortality rates ranged from 3.4 to 28.3 per 1,000 live births in these countries and the UN SDG is a rate of under 12 deaths per 1,000 neonates by 2030. The MOH of supported countries prioritized technical assistance to focus on strengthening the resiliency of newborn and well-baby care systems to address similar future public health emergencies. However, with limited access to confirmatory testing, and the full spectrum of clinical disease from congenital Zika still evolving, it was unclear what percentage of affected pregnant women and their neonates were receiving a diagnosis of Zika, how Zika was impacting neonatal mortality, and whether surviving neonates were being connected to recommended services.

*Essential Care for Every Baby* is one component of the *Helping Babies Survive* (HBS) suite of programs, an initiative of a public–private Global Development including partners such as the American Academy of Pediatrics [6, 7]. HBS curricula are evidence-based, hands-on training programs developed to reduce neonatal mortality in resource-limited environments at a relatively low cost [8]. ECEB specifically teaches health care providers essential newborn care practices to keep all newborns healthy from the time of birth until discharge from the facility, and has been found to improve newborn care practices in other settings [7, 9, 10]. The objective of the ECEB Eastern and Southern Caribbean in-country program was to create a system of sustainable training dissemination that contributes to improving newborn care-related quality indicators in the context of Zika. The aims of this study were to determine if health care providers would have an increase in knowledge about newborn care and signs of congenital Zika after attending an adapted ECEB course which emphasized features of Zika that present in newborns, to determine if skills were retained through the training-of-trainers cascade, and to monitor relevant clinical processes and outcomes at the facility and regional level. We hypothesized that health care providers would demonstrate an increase in newborn care knowledge after attending the workshop regardless of the type of ECEB facilitator. Our objective was to demonstrate improved newborn care at the national and regional level after ECEB courses as part of a set of interventions.

## Methods

The overall project by USAID ASSIST used a combination of different methods. All 134 functional health facilities in four Caribbean countries were included in this work that was intended to expand clinical capacity and improve care of newborns and children potentially affected by Zika. The full project is described elsewhere [10] and the aims included improving timeliness and adherence to recommended clinical care for infants affected by congenital syndrome associated with Zika (CSaZ), improving timeliness and adherence to neurodevelopmental delay screening and referral for children under 5 years old in outpatient clinics, improving newborn care with a focus on evaluation at birth to detect CSaZ, and improving the quality of psyscho-social support services for families affected by Zika. Teams were provided with monthly quality improvement (QI) coaching. Zika clinical care guidelines and provider decision support tools were developed and disseminated. American Academy of Pediatrics (AAP), USAID ASSIST’s partner organization, provided clinical content expertise, including Essential Care for Every Baby (ECEB) training. Performance indicators were selected to monitor improvement, and data were collected and entered into a web-based improvement indicator database to track performance at facility, district, and national level. Four of the ten performance indicators were related to ECEB content, while the others were related to screening, referrals, and clinical care of older children with CSaZ or suspected neurodevelopmental delay. Each component was required for multi-component indicators for it to be considered complete.

**Fig. 1:**
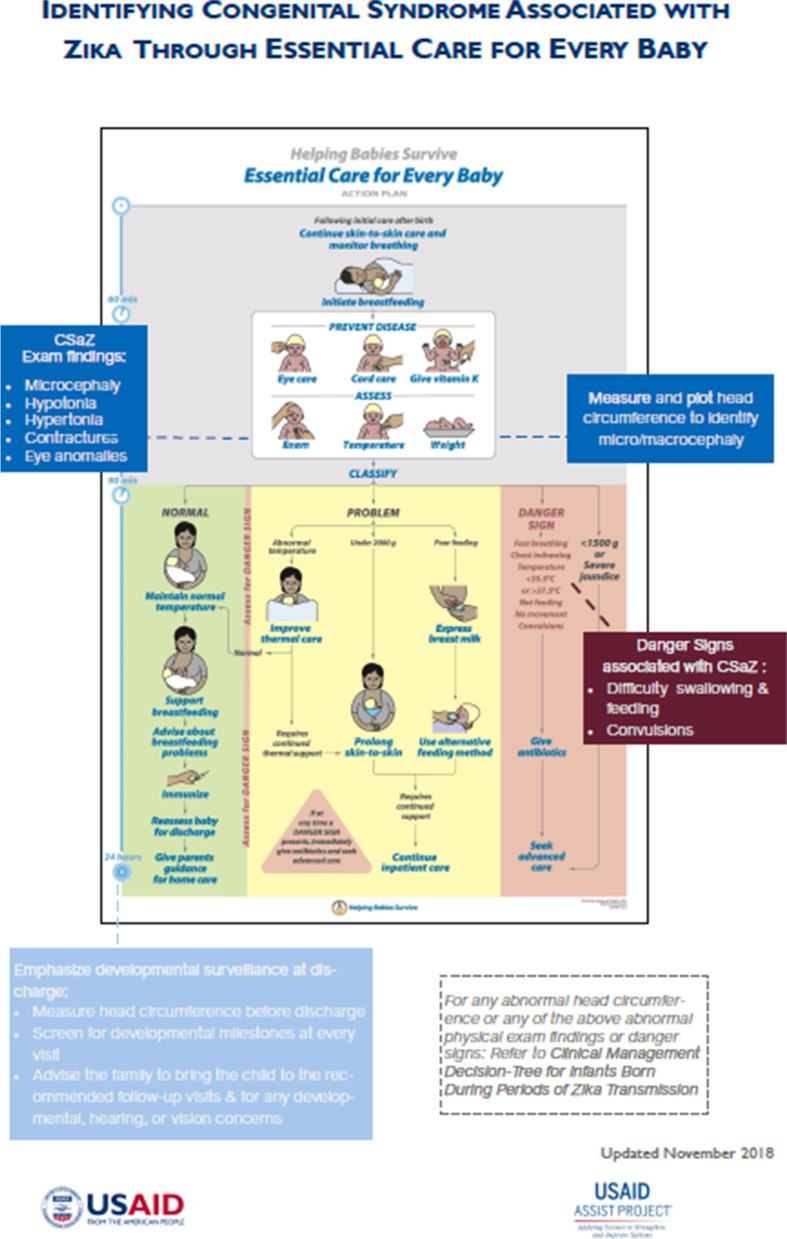
Modified Action Plan. The Essential Care for Every Baby Action Plan was modified to incorporate screening for signs and symptoms of Congenital Syndrome associated with Zika at birth. United States Agency for International Development Applying Science to Strengthen and Improve Systems Project Zika response in Antigua, Dominica, St Kitts and Nevis, and St Vincent and the Grenadines with partnership by the American Academy of Pediatrics, August 2018 to July 2019

**Fig. 2:**
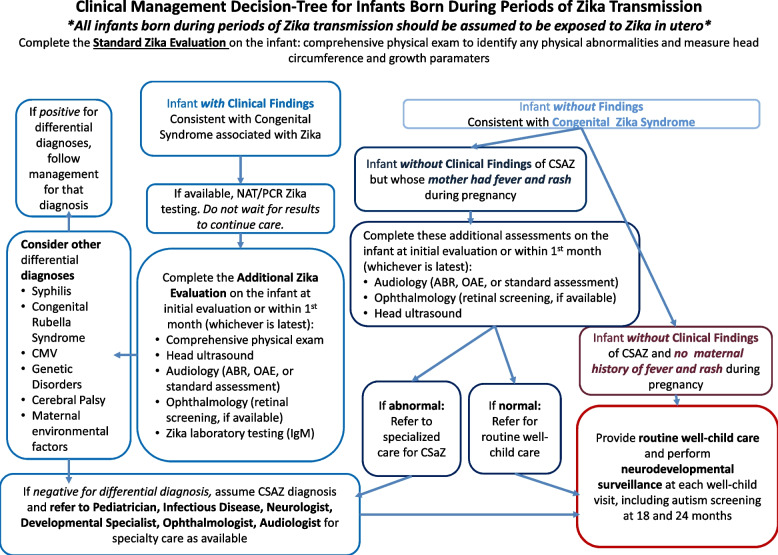
Clinical management decision-tree for infants born during periods of Zika transmission. After formal approval from the Ministry of Health, this management tool was distributed to health care facilities. This was adapted from the Center for Disease Control recommendations within the constraints of the local resource availability. This decision-tree harmonized with Essential Care for Every Baby, so that the tree begins with the Standard Zika Evaluation which was taught in the workshop. The layout, but not the content, was modified for publication. United States Agency for International Development Applying Science to Strengthen and Improve Systems Project Zika response in Antigua, Dominica, St Kitts and Nevis, and St Vincent and the Grenadines with partnership by the American Academy of Pediatrics, August 2018 to July 2019

Beginning in July 2018, USAID ASSIST supported a series of technical assistance visits by the AAP to disseminate ECEB training within the larger scope of work focused on strengthening the clinical capacity in care of newborns and children potentially affected by Zika. ECEB, a skills-based program to teach essential newborn care from birth until discharge, was adapted within this context. ECEB utilizes hands-on learning and practice using a newborn simulator and breastfeeding simulator [11]. Additional educational materials include the Action Plan, flip charts, provider guide, parent guide, a multiple-choice questionnaire “Knowledge Check,” and two Objective Structured Clinical Evaluations (OSCEs). The ECEB knowledge check includes 25 multiple choice questions, and the OSCEs are role-playing, performance-based tests, with a list of skills that need to be demonstrated by the test-taker within a time limit following a verbal prompt. In preparation for the in-country ECEB workshops, the Action Plan was adapted to emphasize screening for signs and symptoms of Zika at birth (Fig. 1). An insert to the provider guide was created and disseminated to all learners at the workshops to focus on facility-level Zika clinical care improvement (see Supplementary material – modified for publication). These adapted ECEB materials harmonized with other job aids provided as part of this project, including standardized anthropometric growth charts for microcephaly screening, a Clinical Management Decision-Tree for Infants Born During Zika Transmission (Fig. 2), and a Signs of Zika at Birth poster. The knowledge check and OSCEs were not altered for this project. Four of the pre-existing questions in the knowledge check are related to difficulties with swallowing or convulsions, which are possible signs of Zika at birth. The “examines baby” task of the OSCE is the opportunity for a provider to note signs of Zika at birth.

With use of these adapted materials and during hands-on training, technical advisors from the AAP emphasized physical exam findings indicative of CSaZ; measurement, plotting and documentation of newborn growth parameters with an emphasis on head circumference; and developmental surveillance after discharge. In each country, in November and December 2018, an AAP technical advisor taught 2–3 ECEB workshops with 6–10 learners each. Three months later, identified in-country ECEB champions (4–7 per workshop) were coached by AAP technical advisors to facilitate additional trainings for naive learners (2–7 per workshop). The in-country ECEB champions were selected by their respective MOHs based on their role within the health care facility and leadership ability. This train-the-trainer model has been used in other HBS programs [12].

At each workshop, a pre- and post- ECEB Knowledge Check and two OSCEs were administered. A repeated measures ANOVA was conducted to evaluate factors associated with significant change in Knowledge Check score. This compared the pre-course Knowledge Check scores and the post-course Knowledge Check scores between subjects who attended an AAP advisor-led workshop and those who attended a local champion-led workshop. This article was written according to Standards for Quality Improvement Reporting Excellence (SQUIRE) guidelines [13].

## Results

Seventy-one health providers were identified to attend an ECEB workshop with Zika emphasis across the four countries (Table 1). Data regarding the total number of health providers and potential audience at each site were not available to the AAP team. Nurse-midwives were the most common type of learner in the AAP led workshop (32/71, 45%), yet fewer learners in the local-champion led workshop were nurse-midwives (4/24, 17%). There was a greater proportion of house officers (physicians-in-training) and physicians in the local champion-led workshops. Local champions were supported in further facilitation of training within the health care facilities.

**Table 1:** Self-described roles of workshop participants

	Nov-Dec 2018 AAP Advisor-Led Workshop n (%)	March 2019 Local Champion-Led Workshop n (%)
Obstetrical House Officers	4 (5)	0
Pediatric House Officers	3 (4)	2 (8)
Nurse-midwives	32 (45)	4 (17)
Staff Nurses	30 (42)	7 (22)
Ward Managers	1 (1)	1 (3)
Physician	1 (1)	2 (8)
Family Nurse Practitioner	0	5 (21)
**Total Learners**	**71**	**24**

Seventy-one health providers were identified to attend an Essential Care for Every Baby workshop with Zika emphasis across four Caribbean countries. The first series of workshops were led by American Academy of Pediatrics (AAP) technical advisors. The second series of workshops were led by local champions, who were supported in further facilitation of training within the health care facilities

In the first series of workshops, knowledge check results improved from an average 19.1 correct answers of 25 questions pre-workshop, to 23.4 correct answers post-workshop (*n* = 70). Twenty-four selected local champions led subsequent workshops, with knowledge check improvement from an average of 20.3 correct answers before the workshop to 23.8 correct answers after workshop completion (*n* = 18). A repeated measures (pre-post) ANOVA with between-subjects factor of workshop leader (AAP vs. local) was conducted (Table 2).

**Table 2:** Repeated measures ANOVA of pre-workshop and post-workshop knowledge check scores

	AAP Advisor-Led Workshop	Local Champion-Led Workshop	
Pre-Workshop Knowledge Check Score (25 Qs)	19.1	20.3	
Post-Workshop Knowledge Check Score (25 Qs)	23.4	23.8	Facilitator effect *p* < 0.001
		Pre-/post- effect: *p* < 0.05	

A repeated measures ANOVA was conducted to evaluate factors associated with significant change in knowledge check score after a series of modified Essential Care for Every Baby workshops in the Caribbean. This compared the pre-course knowledge check scores and the post-course knowledge check scores between subjects who attended an American Academy of Pediatrics (AAP) technical advisor-led workshop and those who attended a local champion-led workshop

This revealed significant pre-post knowledge gains [*F*(1) = 197.9, *p* < 0.001]. There was a significant effect of type of facilitator on knowledge check scores, with a greater improvement when workshops were led by the AAP technical advisor [*F*(1,1) = 4.065, *p* = 0.047]. Qualitative, formative feedback was provided to participants after the OSCE based on individual performance. All participants passed the OSCE. The local champions in two countries also took the knowledge check before and after the workshop that they led. The knowledge check scores prior to their own workshop were lower than the knowledge check scores after their first training.

At each workshop, participants brought feedback that there was a discrepancy between documentation forms at the hospitals and the documentation required per the modified ECEB workshop. The most common inconsistencies were between measuring and documenting temperatures in Fahrenheit versus Celsius, and anthropometric assessment and corresponding documentation with use of a decimal place and standardized growth charts. Participants also had a structured opportunity for feedback at the final technical assistance visit in a focus group. Participants identified barriers to ongoing review and practice of ECEB materials, and specifically named competing clinical demands and limited time availability in a work day.

**Fig. 3:**
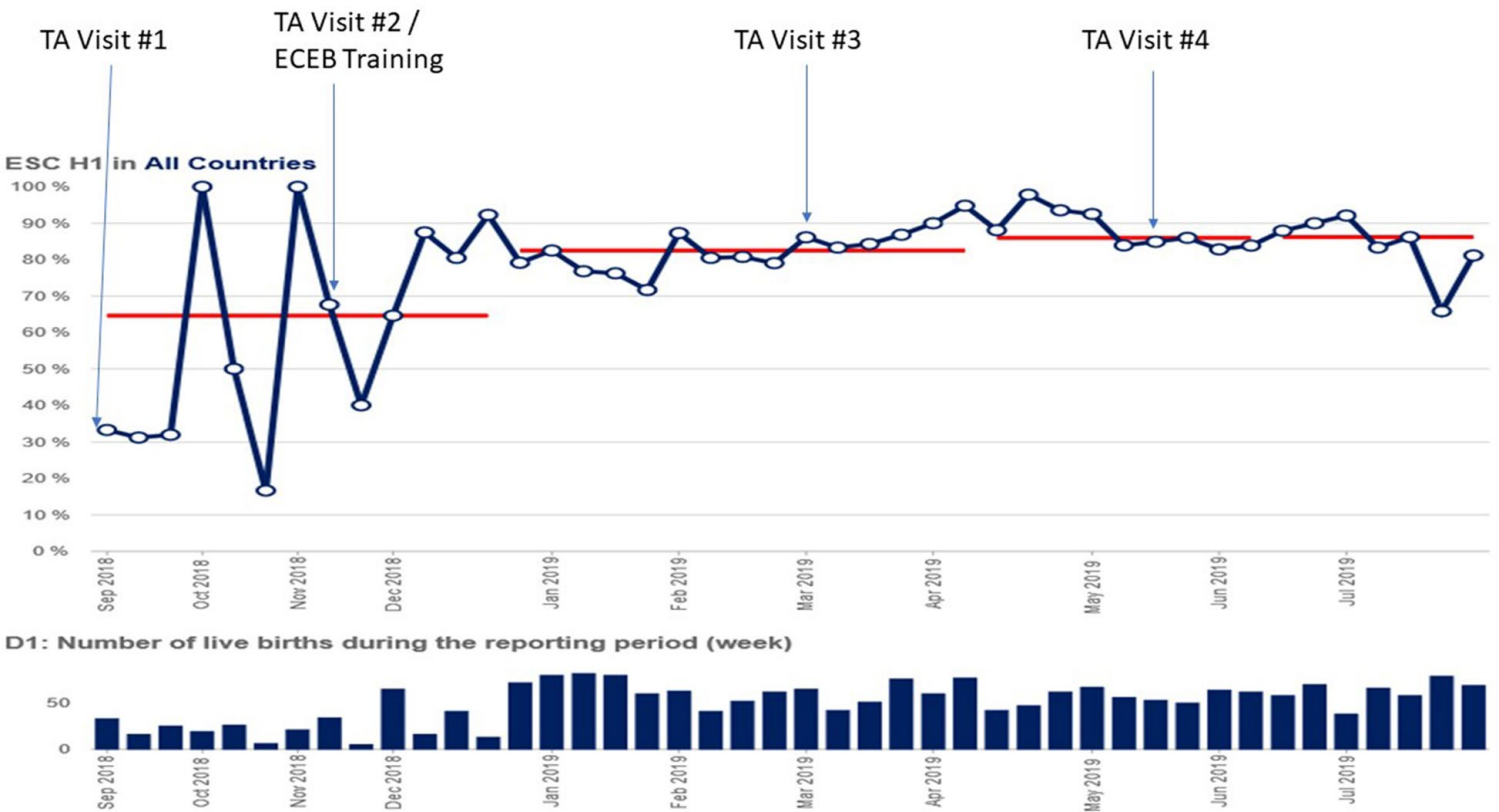
Proportion of newborns receiving essential newborn care interventions (Essential Newborn Care Practices included: 1) breastfed within one hour of birth; 2) immediate skin to skin contact with their mothers within the first hour after birth; 3) Vitamin K administration; and 4) eye care before hospital discharge.) in all functional hospitals of Antigua and Barbuda, St Kitts and Nevis, St Vincent and Grenadines and Dominica. United States Agency for International Development Applying Science to Strengthen and Improve Systems (USAID ASSIST) Project Zika response in Antigua, Dominica, St Kitts and Nevis, and St Vincent and the Grenadines with partnership by the American Academy of Pediatrics (AAP), August 2018 to July 2019. Technical assistance (TA) visits are annotated. The second TA visit included the first Essential Care for Every Baby (ECEB) workshops led by the AAP

Over the year, some outcome and process measures improved in all four countries (Table 3). Certain indicators related to ECEB practices demonstrated significant changes and midline shift on the run chart. This included proportion of newborns receiving essential newborn care practices in the hospital (Fig. 3), appropriate screening and classification of microcephaly, documentation of essential assessment practices in the hospital, and exclusively breastfeeding at hospital discharge. The ECEB training was part of the larger clinical capacity building that also emphasized team-based problem solving and health system strengthening activities.

**Table 3:** De-identified change in performance indicators in all functional hospitals of Antigua and Barbuda, St Kitts and Nevis, St Vincent and Grenadines and Dominica, before and after United States Agency for International Development Applying Science to Strengthen and Improve Systems (USAID ASSIST) Project Zika response with partnership by the American Academy of Pediatrics (AAP). Performance data were collected and entered into a web-based improvement indicator database, allowing performance tracking over time at the facility, district, and national levels in run charts that were evaluated by statistical process control for significant improvement

Performance Indicators	Country	Baseline (2018)	End line (Nov. 2019)^a^
Percent of newborns receiving all essential newborn care interventions before discharge	A	12%	100%
	B	90%	79%
	C	25%	93%
	D	21%	64%
Percent of newborns who were appropriately screened for microcephaly	A	100%	98%
	B	33%	48%
	C	0%	100%
	D	1%	63%
Percent of newborns in postnatal care wards or areas in the health facility with all essential assessment practices	A	33%	100%
	B	80%	37%
	C	84%	100%
	D	73%	100%
Percent of children with exclusive breastfeeding at discharge	A	65%	88%
	B	100%	45%
	C	54%	86%
	D	82%	100%

^a^ Latest hospital data is from November 2019 for 3 countries. For one country only, data are current as of August 2019; hospital data were not entered since August due to staff shortages and the transfer of the Records Department to the new hospital which made it difficult to access the patient records

## Discussion

This project demonstrated that ECEB workshops improved objective knowledge of health care providers, as evidenced by a significant difference in pre- to post-knowledge check scores. Although there was a statistically significant difference in the pre- to post- knowledge check scores dependent on the type of facilitator leading the workshop, the magnitude of the difference was small, and the post-workshop scores with both types of facilitators were very similar. It is not surprising that experienced ECEB facilitators may be slightly more effective than novice facilitators, particularly when there has been a gap in their learning of the material and then their first time facilitating the material, emphasizing the need for immediate implementation to reinforce the concepts learned. A limitation of this study is that the groups of learners were not necessarily comparable, as described in Table 1. However, our results indicate that hands-on, in-the-moment training of trainers can effectively coach facilitators and ensure fidelity to the training methods and content, so that the same final results are achieved.

In one country, the local champions also took the knowledge check before and after the workshop they led. The knowledge check scores prior to leading their own workshop were lower than their knowledge check scores following their initial training. While this was not done at each site, precluding firm conclusions, these results do fit with what is commonly known about knowledge and skills after an initial workshop—they decline if not practiced regularly. There is evidence to support the use of low dose, high frequency practice to improve skills retention [14–16]. Ideas generated by participants on how to implement such practice included using the “review key knowledge” section of each page of the provider guide to frequently test skills with a partner, and incorporating ECEB into pre-service education of nursing trainees and house staff officers by dividing the course into hour-long sessions. Finally, teaching new providers both disseminates the information and reinforces the knowledge of the local champion leading the educational session. ECEB clinical practices were continuously measured by the QI teams and reinforced during coaching visits and learning sessions to support knowledge retention and translation into clinical practice.

Logistical planning was crucial for the successful dissemination of training. One common theme of discussion at all sites and at sequential workshops included the importance of keeping the materials easily accessible in a designated location. It is universally acknowledged that health care workers have busy clinical schedules, and so it is important to improve accessibility to the written materials and simulators to facilitate their frequent use as job aids and training tools. A simple intervention would be to identify a place to store the materials. In some settings, teams created a protocol of “checking out” the materials to borrow and return. In most settings, the Action Plan poster (Fig. 1) and Signs of Zika at birth poster were strategically hung near the maternity area so that health care providers could easily, and frequently, reference them. Improving accessibility helped ameliorate the barrier of competing clinical demands and the opportunity cost of spending time on reviewing these materials instead of another clinical task.

Ongoing monitoring of performance indicators demonstrated improvement in all four countries, with the ECEB workshop as just one piece of the wide scale training and implementation of novel tools. Thus, improvement of performance indicators most likely resulted from a combination of interventions. A benefit of using ECEB training as a complement to the other training and service delivery in the project allowed for participants to synthesize their new knowledge and skills, resulting in improvement in performance indicators related to breastfeeding, and not just Zika identification. It is possible that solely providing ECEB training without the other wide scale training would result in a lower magnitude of change in the performance indicators. The uptake of the skills reinforced by ECEB are best demonstrated in the run charts for indicators specific to the Zika-specific ECEB workshop.

It is encouraging that naïve facilitators can be coached to become local experts to continue dissemination of training. ECEB is a model for cascade training that incorporates essential newborn practices with the opportunity to adapt to the context of local practices and respond to public health emergencies. Many of the signs and symptoms screened for in our adapted curricula are shared with other congenital conditions, such as “TORCH” infections [17]. The emphasis on accurate performance and recording of newborn physical and growth parameters will aid in earlier identification of other common syndromes and sequences. We found that key components of successful cascade training included multilevel support, appropriate identification of local champions, inclusion of health care providers who served diverse roles in the same system, strategic planning for training dissemination, and adaptation of the program materials to the local context.

Since this intervention was completed, the World Health Organization (WHO) has adapted the Helping Babies Breathe suite of programs into their new Essential Newborn Care (ENC) Course, 2^nd^ Edition, which was released as an interim version in April 2022, available for download on the WHO website. ENC-1 consists of immediate assessment of the newly born infant, including resuscitation and care through one hour after birth. ENC-2 consists of care of infant from where ENC-1 ends and through discharge. ENC-1 and ENC-2, which replicate much of the HBB and ECEB content, also align with WHO standards for maternal and newborn care, and incorporate infection prevention practices, baby-friendly hospital practices, nurturing care, birth defect surveillance and quality improvement principles.

## Conclusion

ECEB can be adapted to local settings and to specifically address concerns, such as ongoing Zika transmission. These changes can incorporate important local practices, causes of neonatal morbidity and mortality, and differing healthcare system structures. Pre-post workshop knowledge check scores improved with facilitation by both AAP technical advisors and local providers. The Helping Babies Survive creators recognize that it is imperative to build sustainability into a program, and teaching quality improvement methodology is one strategy that promotes scaling up. Continued mentorship and skills building by trained facilitators is important to solidify their knowledge and sustainably strengthen capacity of newborn care providers in these countries.

## Supplementary Information

**Additional file 1.** 

## Data Availability

There are two datasets used in the development of this manuscript. One dataset is included in the Supplemental File, which includes the test performance of participants in the Essential Care for Every Baby program. The other datasets, including performance of the countries and region on Essential Newborn Care in Fig. 3, are intellectual property of the United States Agency for International Development Applying Science to Strengthen and Improve Systems Project. Upon reasonable request, the authors will help an interested inquirer access this data. Please contact the corresponding author Kera McNelis at kera.mcnelis@cchmc.org.

## Abbreviations

AAP: American Academy of Pediatrics
ECEB: Essential Care for Every Baby
HBS: Helping Babies Survive
MOH: Ministry of Health
OSCE: Objective Structured Clinical Exam
QI: Quality Improvement
SDG: Sustainable Development Goals
USAID ASSIST: United States Agency for International Development Applying Science to Strengthen and Improve Systems
