# Supplementary material for: Adaptation of Essential Care for Every Baby educational program to improve infant outcomes in the context of Zika
Source: BMC Pediatr. 2022 Nov 21;22:669. doi: 10.1186/s12887-022-03710-7 (PMC9677636; doi:10.1186/s12887-022-03710-7)
Supplement: Supplementary file 1 — Additional file 1. [file 12887_2022_3710_MOESM1_ESM.zip › Zika_modified_provider_guide_insert.pdf]

Essential Care for Every Baby in the Age of Zika
Provider Guide Insert

Improve care in your facility for Zika care—

Examine the baby (page 15)

To Improve care in your facility:

◊ How can you make it easier to examine all babies in your facility by 90 minutes of age and record the results of the examination?

◊ Discuss the answers to this question with other providers and leaders in your facility. The following questions may help you understand what prevents you from performing this action as recommended.

Suggested questions to identify potential problems:

◊ Who examines babies in your facility?

◊ Is there adequate light where you examine babies?

◊ Are there forms on which to record the results of examinations?

Additional question(s) to consider:

◊ How can you make sure that all babies in your facility have a head circumference measured to the nearest tenth and that this measurement is recorded in the health record?

◊ How do you document the newborn exam and if there are any abnormalities or concerns?

Give parents guidance for home care (page 35)

To Improve care in your facility:

◊ How could you ensure that all parents are given guidance for home care?

◊ Discuss the answers to this question with other providers and leaders in your facility. The following questions may help you understand what prevents you from performing this action as recommended

Suggested questions to identify potential problems:

◊ • Who is responsible for preparing parents for the care of their baby at home?

◊ • Is there a guide or other resource to remind parents about home care?

◊ • Where do parents seek care for their baby if they observe a Danger Sign?

Additional question(s) to consider:

◊ How could you ensure that all babies who have evidence of congenital Zika syndrome are appropriately referred?
